# The Importance of Testing Multiple Environmental Factors in Legume–Insect Research: Replication, Reviewers, and Rebuttal

**DOI:** 10.3389/fpls.2016.00489

**Published:** 2016-04-22

**Authors:** Scott N. Johnson, Andrew N. Gherlenda, Adam Frew, James M. W. Ryalls

**Affiliations:** Hawkesbury Institute for the Environment, Western Sydney UniversityPenrith, NSW, Australia

**Keywords:** atmospheric change, biological nitrogen fixation, climate change, insect–plant interactions, legumes, pastures

## The case for testing multiple environmental factors

Investigating the impacts of predicted changes in our atmosphere and climate change on insect–plant interactions is a widely pursued area of research. To date, the majority of experimental studies have tested the impacts of single environmental factors on insect–plant interactions, but meta-analyses have clearly illustrated the importance of investigating multiple factors in tandem (Zvereva and Kozlov, 2006; Robinson et al., 2012). In particular, environmental change factors often interact with each other which can either strengthen or mitigate the effects of environmental factors acting alone (Robinson et al., 2012). For example, the positive effects of elevated atmospheric carbon dioxide concentrations (e[CO_2_]) on plant growth are stronger under high nitrogen (N) conditions compared to low N conditions (+32 and +19%, respectively; Robinson et al., 2012). Likewise, from the limited number of studies available, Robinson et al. (2012) showed that e[CO_2_] had different impacts on plant nitrogen, plant biomass, and secondary metabolites under elevated air temperature (eT) conditions. This does not invalidate single factor studies, of which we have published numerous examples, but this is an important consideration for making realistic predictions about how plants and insects will respond to future climates (Facey et al., 2014).

## Legume–insect interactions

A key feature of legumes is their capacity for biological nitrogen fixation (BNF), which they accomplish via symbiotic relationships with soil bacteria which associate with the plant in discrete root nodules. Given that insect herbivores are frequently nitrogen limited (Mattson, 1980), concentrations of N in legumes derived from BNF are likely to be crucial determinants of plant–herbivore interactions. Legumes differ markedly from non-legume plants in their responses to environmental change because BNF is often significantly affected (Robinson et al., 2012). Moreover, e[CO_2_] and eT appear to have contrasting effects on BNF; e[CO_2_] tends to promote BNF via several mechanisms (Soussana and Hartwig, 1996), including larger numbers of N_2_-fixing symbiotic bacteria in the rhizosphere (Schortemeyer et al., 1996), increased nodulation (Ryle and Powell, 1992) and enhanced nitrogenase activity (Norby, 1987). In contrast, eT tends to have an inhibitory effect on BNF because of the low tolerance of N_2_-fixing bacteria to higher temperatures (Zahran, 1999; Whittington et al., 2013). These generalizations are, of course, contingent on nutrient availability in the soil (e.g., Edwards et al., 2006).

Given this, one might assume that e[CO_2_] and eT might have contrasting impacts on insect herbivores of legumes since they affect nitrogen concentrations in the plant tissues in a divergent manner. This seems to be the case, with e[CO_2_] either having no adverse effects (e.g., Karowe and Migliaccio, 2011) or, more often, a beneficial impact on herbivore performance (e.g., Johnson and McNicol, 2010), particularly for aphids (Guo et al., 2013, 2014; Johnson et al., 2014). However, our recent work with lucerne (*Medicago sativa*) has shown that the positive impacts of e[CO_2_] on pea aphids (*Acyrthosiphon pisum*) were negated under eT because eT caused decreases in nodulation and amino acid concentrations in the foliage (Ryalls et al., 2013, 2015). Testing multiple environmental factors, including soil nutrients, therefore seems to be particularly relevant for investigations into how legume herbivores will respond to atmospheric and climate change research.

## The challenges: replication and reviewers

Why are there so few multi-factorial experiments in climate change research? Put simply, constraints on replication are the biggest obstacles faced by investigators. Pseudoreplication (a term first coined in Hurlbert, 1984) is particularly common in climate change research (Newman et al., 2011). For example, 49 of the 110 climate change studies reviewed by Wernberg et al. (2012) had pseudoreplication issues. This usually arises because when environmental factors are applied to controlled chambers, glasshouses, or FACE (Free Air CO_2_ Enrichment) rings, the unit of replication for those treatments is the chamber, greenhouse, or ring, respectively (Lindroth and Raffa, in press). Subunits (e.g., individual plants) are not independently subjected to the treatment, and therefore not true replicates. As a result, statistical tests are based on artificially high degrees of freedom, resulting in a larger F statistic, potentially leading to type I errors (i.e., false positives; Lindroth and Raffa, in press). For this reason, many reviewers for scientific journals automatically reject manuscripts if any part of an experiment is pseudoreplicated without necessarily considering whether the biological conclusions of the study are really compromised by pseudoreplication (Davies and Gray, 2015). This is possibly an overzealous interpretation of the case by Hurlbert (1984), the authority on the subject, who states that “there should be no automatic rejection of [such] experiments” (Hurlbert, 2004). In a recent and comprehensive article, Davies and Gray argue convincingly that reviewers erroneously and dogmatically reject papers that have pseudoreplication issues which is slowing the pace of ecological research. While Davies and Gray (2015) focussed on non-manipulative experiments in natural systems, many of the points were germane to multi-factorial climate change research. In particular, many contemporary statistical tests, such as nested designs and random/mixed effect models, account for the lack of independence between pseudoreplicates so may help in some cases (Chaves, 2010; Leather et al., 2014; Davies and Gray, 2015). Of course, such statistical approaches could only help where a treatment combination was repeated in more than one chamber, glasshouse, or FACE ring.

## Comparing experimental approaches—potential for rebuttal?

How do researchers attempt to overcome the pseudoreplication problem experimentally? The simplest way is to avoid it altogether by fully replicating environmental treatments. However, using even the bare minimum of replicates (e.g., *N* = 4) would require 16 separate chambers, glasshouses, or rings for an e[CO_2_] × eT experiment. Many researchers cannot readily access this number of identical facilities or monopolize them for that matter. Repeating the experiment several times and using experimental run as the source of replication is another approach (e.g., Johnson et al., 2011), but this can be logistically demanding in time and cost. Even when fully replicated, the degrees of freedom in these studies are often so low that they are susceptible to type II errors, whereby “real responses” are not statistically detected (e.g., the “false negative”).

Another approach that researchers sometimes use is “chamber swapping”, whereby experimental units (e.g., plants) are moved within, and then between, chambers with attendant changes in environmental conditions (e.g., Bezemer et al., 1998). This does not eliminate pseudoreplication, but rather serves to minimize its effects by equalizing any unintended “chamber effects” across all experimental units. While this approach might be criticized because chamber effects might affect plants differently during different stages of their development (Potvin and Tardif, 1988), researchers have addressed this by staggering experiments so plants are exposed to particular chambers at the same stage of development (e.g., Vuorinen et al., 2004a,b).

**Figure 1:**
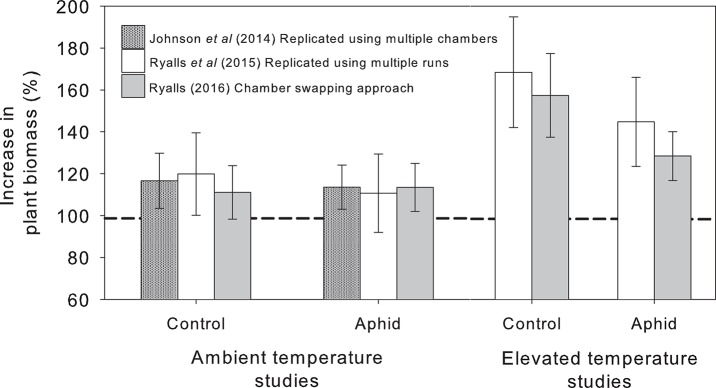
**Relative change in plant biomass at elevated [CO_**2**_] compared to plants grown at ambient [CO_**2**_], indicated with the dashed line, with and without (control) aphids (mean ± ***S.E.*** shown)**. Data from three experiments using replication with multiple chambers (Johnson et al., 2014) and multiple experimental runs (Ryalls et al., 2015) compared with the “chamber swapping” approach (Ryalls, 2016). All experiments used the same cultivar (Sequel) and similar levels of [CO_2_] (400 vs. 600–640 ppm) and temperature (25–26 vs. 30°C).

How do results from a “chamber swapping” experiment compare with replicated experiments? We can answer this question, in part, using three comparable published studies that examined the impacts of environmental change on interactions between lucerne and the pea aphid. One experiment was replicated using multiple chambers (Johnson et al., 2014), one replicated using multiple experimental runs (Ryalls et al., 2015) and one adopted the chamber swapping approach (Ryalls, 2016). The first of these only examined e[CO_2_], whereas the other two experiments also included eT. Figure 1 shows the increase in dry mass of plants (with and without aphids) grown under e[CO_2_] and eT relative to plants grown under ambient conditions. This response was selected for comparison since it was evidently measured the same way in each experiment. Despite using very different approaches, in most cases we obtained very similar responses whether the experiment was fully replicated or conducted with regular chamber swaps (c. every 10 days). Analysis of variance suggested that study type had little impact on the response we measured [*F*_(2, 219)_ = 0.20, *P* = 0.82]. This is a crude comparison, but it is reassuring that we obtained similar data and reached identical conclusions using the chamber swapping approach.

## Conclusions and recommendations

While incorporation of multiple environmental factors is desirable in many climate change studies of plant–herbivore interactions (clearly advocated by Robinson et al., 2012), we argue here that it is especially relevant to legume–insect research. Nitrogen status in legumes is shaped by BNF, which is highly affected by atmospheric and climatic change, often in divergent directions. This will inevitably affect legume quality for herbivores (i.e., especially primary metabolites, but possibly secondary metabolites too), and likely affect herbivore abundance and performance. Nonetheless, experimental manipulation of multiple factors is challenging and prone to pseudoreplication. “Chamber swapping” does not eliminate this problem, but it appears to minimize “chamber effects” and give comparable results to fully replicated experiments—at least in the lucerne-aphid system. We recommend that researchers working in other systems also take a cautious approach with regard to careful replication until they can develop confidence that their observed effects are real and repeatable. The statistical significance of numerical differences remain inflated, however, so it would be judicious to treat any marginally significant results with caution and rather interpret effect sizes rather than *P*-values *per se* (see discussion by Ellison et al., 2014). Davies and Gray (2015) make the similar arguments and suggest that conclusions can be phrased as new hypotheses if necessary. In conclusion, we agree with Newman et al. (2011) on this issue that “as long as authors are clear about the use of pseudoreplicates, and the readers appreciate the potential problems interpreting such results, then such studies are valuable despite their pseudoreplication.”

## Author contributions

SJ conceived and drafted the article with significant intellectual input from all authors. JR conducted the majority of the experimental work described in the article, with SJ, AG, and AF overseeing collection of further data used in Figure 1.

### Conflict of interest statement

The authors declare that the research was conducted in the absence of any commercial or financial relationships that could be construed as a potential conflict of interest.
